# Remote control of glucose homeostasis *in vivo* using photopharmacology

**DOI:** 10.1038/s41598-017-00397-0

**Published:** 2017-03-22

**Authors:** Zenobia B. Mehta, Natalie R. Johnston, Marie-Sophie Nguyen-Tu, Johannes Broichhagen, Peter Schultz, Dean P. Larner, Isabelle Leclerc, Dirk Trauner, Guy A. Rutter, David J. Hodson

**Affiliations:** 1 Section of Cell Biology and Functional Genomics, Department of Medicine, Imperial College London, London, W12 0NN UK; 2 Department of Chemistry and Center for Integrated Protein Science, LMU Munich, Munich, Germany; 3 Institute of Metabolism and Systems Research (IMSR) and Centre of Membrane Proteins and Receptors (COMPARE), University of Birmingham, Edgbaston, B15 2TT UK; 4 Centre for Endocrinology, Diabetes and Metabolism (CEDAM), Birmingham Health Partners, Birmingham, B15 2TH UK; 5 Max-Planck Institute for Medical Research, Jahnstr. 29, 69120 Heidelberg, Germany

## Abstract

Photopharmacology describes the use of light to precisely deliver drug activity in space and time. Such approaches promise to improve drug specificity by reducing off-target effects. As a proof-of-concept, we have subjected the fourth generation photoswitchable sulfonylurea JB253 to comprehensive toxicology assessment, including mutagenicity and maximum/repeated tolerated dose studies, as well as *in vivo* testing in rodents. Here, we show that JB253 is well-tolerated with minimal mutagenicity and can be used to optically-control glucose homeostasis in anesthetized mice following delivery of blue light to the pancreas. These studies provide the first demonstration that photopharmacology may one day be applicable to the light-guided treatment of type 2 diabetes and other metabolic disease states *in vivo* in humans.

## Introduction

The production of drugs with improved safety profiles remains of paramount importance to ensure healthy aging across lifespan. A significant cause of drug toxicity is off-target effects on cognate receptors expressed outside of the tissue of interest^1^. This can impact disease management, especially for chronic diseases such as type 2 diabetes (T2D) where the aim is to maintain normal glucose homeostasis for long periods with as few side effects as possible.

To this end, sulfonylureas have been used for the treatment of T2D for the past half century, since they bind the SUR1 subunit of the ATP-sensitive potassium (K_ATP_) channel to potently induce insulin release^2–4^. However, they can cause dangerous hypoglycemic swings, and also block K_ATP_ channels in the heart to increase cardiovascular mortality risk^5, 6^. Nonetheless, they are among the most potent insulin secretaogues studied to date^7^, their safety profile is well-understood due to data from large patient cohorts, and they are inexpensive (*e.g.* compared to incretins). As such, sulfonylureas make excellent candidates for further refinement.

Recently, we described a synthetic approach to install azobenzene photoresponsive moieties onto the backbone of glimepiride, a third generation sulfonylurea, in a process termed “azologization”^8^. The resulting compounds, JB253 and JB558, are photochromic ligands (PCLs) that undergo isomerization in response to illumination with discrete wavelengths. This changes their interactions with the SUR1 subunit of the K_ATP_ channel, affording reversible optical control over beta cell K^+^ conductance, Ca^2+^ fluxes and insulin release^9, 10^. Thus, JB253 and JB558 are fourth generation or so-called “photoswitchable” sulfonylureas (IUPHAR/BPS ligand id: 7787).

Importantly, such photopharmacological approaches allow drug activity to be targeted using light^11, 12^. Indeed, photopharmacology has been used to restore visual responses in blind mice^13^, produce “resistance-resistant” antibiotics^14, 15^, generate targeted chemotherapeutics^16, 17^, and alter behavior in zebrafish^18^. Moreover, the applicability of photopharmacology to T2D has been shown not only through the production of light-activated sulfonylureas^9, 10^, but also incretins^19^ and positive allosteric modulators of the glucagon-like peptide-1 receptor^20^.

Although significant technical hurdles remain, notably concerning light delivery, photopharmacology may improve T2D treatment by delivering drug activity specifically to the pancreas only when required, for example in the immediate postprandial period when insulin release is thought to be most beneficial^21^. This would help alleviate problems due to off-target effects (*e.g.* in the heart^22, 23^), as well as those caused by prolonged activity profiles (*e.g.* beta cell exhaustion or “exocytotoxicity”^24^). Despite this, *in vivo* testing of PCLs has to date been restricted to lower vertebrates^18, 25, 26^ or *via* topical application of compound to peripheral sites (*i.e*. the eye)^13^, and toxicology data following systemic delivery is lacking.

In the present study, we therefore sought to assess the safety profile of JB253, as well as demonstrate its *in vivo* efficacy for the optical control of glucose homeostasis in anesthetized mice following illumination of the pancreas with blue light.

## Results

### JB253 bulk synthesis

**Figure 1:**
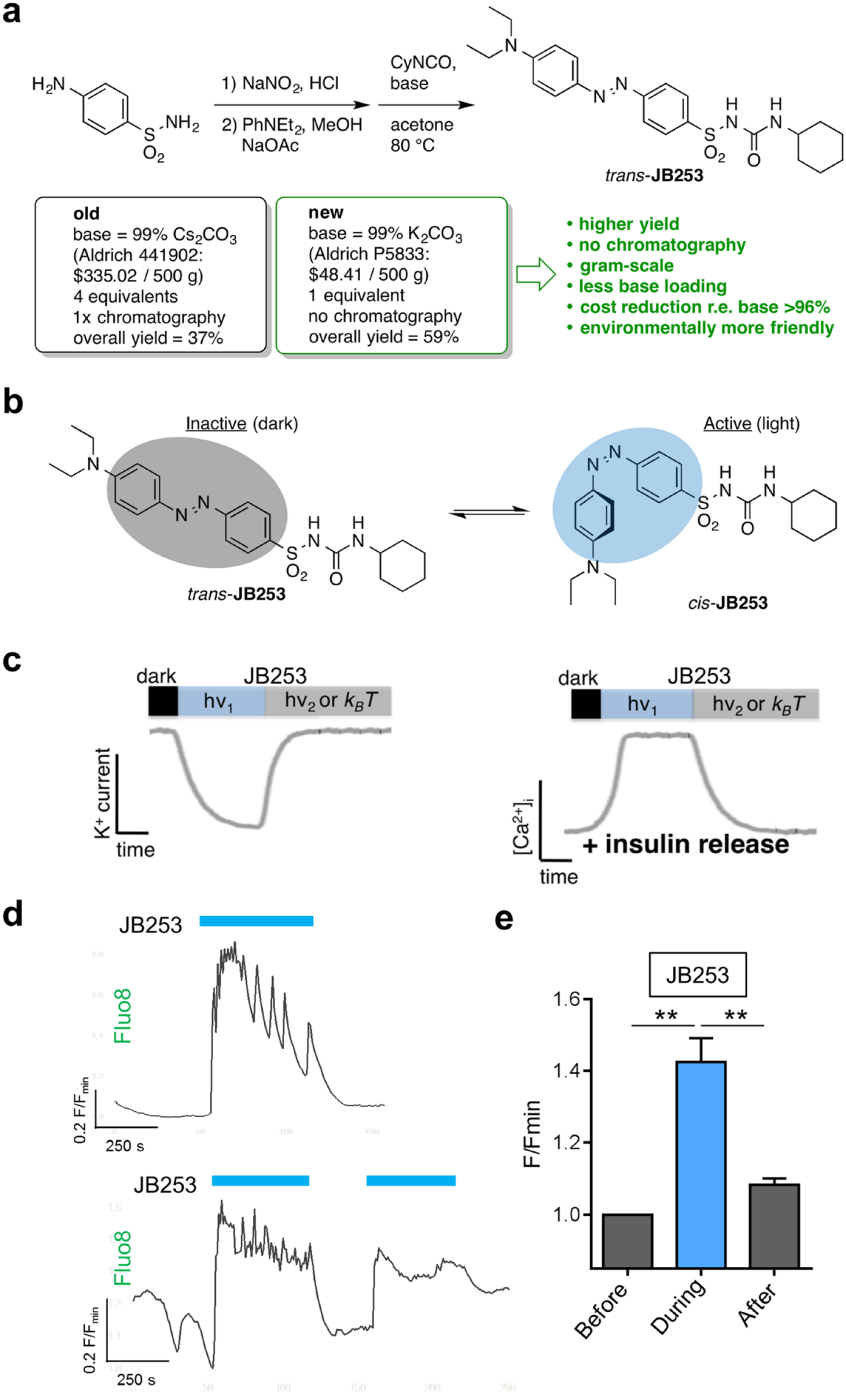
JB253 bulk synthesis. (**a**) An alternative synthetic route affords JB253 *via* crystallization and no chromatography from 1 equivalent of base in the second step. This can be easily performed on the gram-scale, is more environmentally-friendly and allows significant cost reductions and enhanced yield. (**b**) *trans*-JB253 (dark) is inactive, whereas illumination with blue light leads to *cis-*isomerization and activation. (**c**) Schematic showing that exogenously-applied JB253 closes K_ATP_ channels, leading to membrane depolarization, Ca^2+^ influx and insulin release. This process can be switched back and forth using light of the appropriate wavelength, or the dark (hv_1_ = photon energy needed for isomerization to achieve channel closure; hv_2_ = photon energy needed for isomerization to achieve channel opening, also achievable by thermal relaxation *k*_*B*_ *T*) (see also ref. 10). (**d**) Representative traces showing that JB253 produced using bulk synthesis allows the reversible optical control of Ca^2+^ fluxes *in vitro* in isolated pancreatic islets incubated with 8 mm *D*-glucose. (**e**) As for (**d**), but summary bar graph showing Ca^2+^ levels before, during and after illumination with blue light (representative traces; *n* = 9 recordings from 3 animals). **P < 0.01; repeated measures one-way ANOVA (Bonferroni’s posthoc test). Values represent mean + SEM.

To allow the production of gram-scale quantities of JB253, a new synthetic route was devised. Commencing with sulfanilamide, diazotization was carried out as previously reported^10^ and the JB253 precursor (*E*)-4-((4-(diethylamino)phenyl)-diazenyl)benzenesulfonamide collected as a crude compound by extraction. Sulfonylurea installation was then optimized using one equivalent of potassium carbonate (*cf* four equivalents of cesium carbonate previously), before purification *via* crystallization to access JB253 in high purity and 59% overall yield (*cf* 37% previously) (Fig. 1a). To date, >100 g of JB253 has been produced using this method. In response to dark and light, JB253 adopts its *trans* (inactive)- and *cis* (active)- isomer, respectively (Fig. 1b). This allows the reversible blockade of K_ATP_ channels in pancreatic beta cells, leading to increases in Ca^2+^ conductance and Ca^2+^-dependent exocytosis of insulin granules (see schematic in Fig. 1c)^9, 10^. The new synthetic route did not alter photoswitching properties, since reversible optical control could be afforded over Ca^2+^ fluxes in intact islets of Langerhans treated with bulk-produced JB253 (Fig. 1d and e).

### Mutagenicity testing

JB253 (5–5000 µg) was tested for mutagenic activity in *Salmonella typhimurium* TA 1535, TA 100, TA 1537, TA 98 and *E. coli* WP2uvrA strains in the absence and presence of rat liver S9 preparation (Supplementary Tables 1–4). Mutation frequencies were doubled *versus* control in the absence of S9 mix in TA 1537 at 500 µg per plate (Supplementary Table 1). Similar results were obtained in the presence of S9 mix in strains TA 1535 at 5, 50 and 1667 µg per plate, and TA 1537 at 500 and 5000 µg per plate (Supplementary Table 3). However, mean counts were all below 20 and so not considered mutagenic^27^. By contrast, in the presence of S9 mix, mutagenic activity was observed in strain TA 1537 at concentrations of 17 and 50 µg per plate (Supplementary Table 3), and in the *Escherichia coli* strain at concentrations of 500 to 5000 µg per plate (Supplementary Table 4).

### Toxicology testing

**Figure 2:**
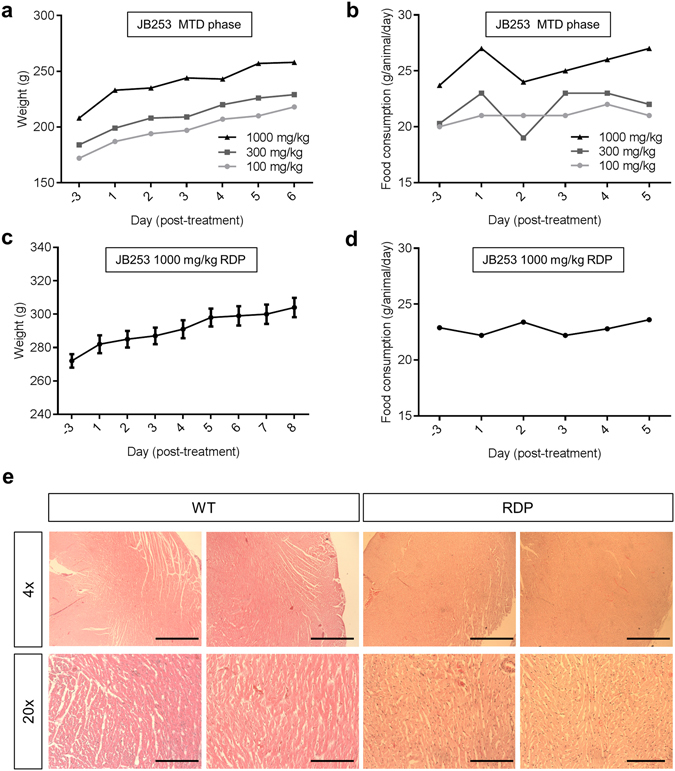
JB253 toxicology studies. (**a**) An animal receiving escalating doses (100–1000 mg/kg) during the maximum tolerated dose (MTD) phase did not show reductions in body weight). (**b**) As for (**a**), but food consumption (average chow consumed; *n* = 4 animals grouped). (**c**) As for (**a**), but showing no changes in weight during the repeated dose phase (RDP) at the maximum dose (1000 mg/kg) (*n* = 5 animals). (**d**) As for (**b**), but showing no changes in food consumption per animal during the RDP at the maximum dose (1000 mg/kg) (average chow consumed; *n* = 5 animals grouped). (**e**) Histopathological examination of hearts from wild-type (WT) and RDP animals was inconclusive with apparently normal cardiomyocyte morphology and no evidence of toxicity (representative images shown; *n* = 3–4 animals) (scale bar top = 500 µm; scale bar bottom = 100 µm). Animal numbers were set according to the UK Home Office, and in line with the OECD Mutual Acceptance of Data Agreement. Values represent mean ± SEM where grouped values are considered.

JB253 appeared to be well-tolerated when administered *via* oral gavage, with no adverse effects reported in animals during both the maximum tolerated dose (MTD) (100–1000 mg/kg) and repeated dose (1000 mg/kg for 7 days) phases. Body weight and food consumption (Fig. 2a–d) were maintained throughout. The only clinical observation was the presence of red feces, which is likely a consequence of the compound pigment (bright red), and no JB253-related gross pathology findings were found upon necropsy. No mortality was reported during the study. Weights of the heart, brain and kidney were no different to reference values from animals of the same sex and age (Table 1). Moreover, histopathological examination of hearts from rats in the repeated dose phase revealed normal cardiomyocyte morphology, with no obvious signs of toxicity such as fiber disarray, inflammatory infiltrate, pyknosis or deposits (*e.g.* amyloid) compared to wild-type controls (Fig. 2e).

**Table 1:** Organ weights of male animals receiving 1000 mg/day JB253 for 7 days *versus* reference values.

Animal No.	Brain (g)	Heart (g)	Kidney (g)
4001	2.01	1.06	2.04
4002	1.84	0.95	1.89
4003	1.92	0.91	2.09
4004	1.96	1.08	2.10
4005	1.97	1.02	2.17
**Mean**	1.94	1.00	2.06
**SD**	0.06	0.06	0.10
**Reference**	1.98	1.01	2.15
**SD**	0.05	0.04	0.11

### Compound cleavage

**Figure 3:**
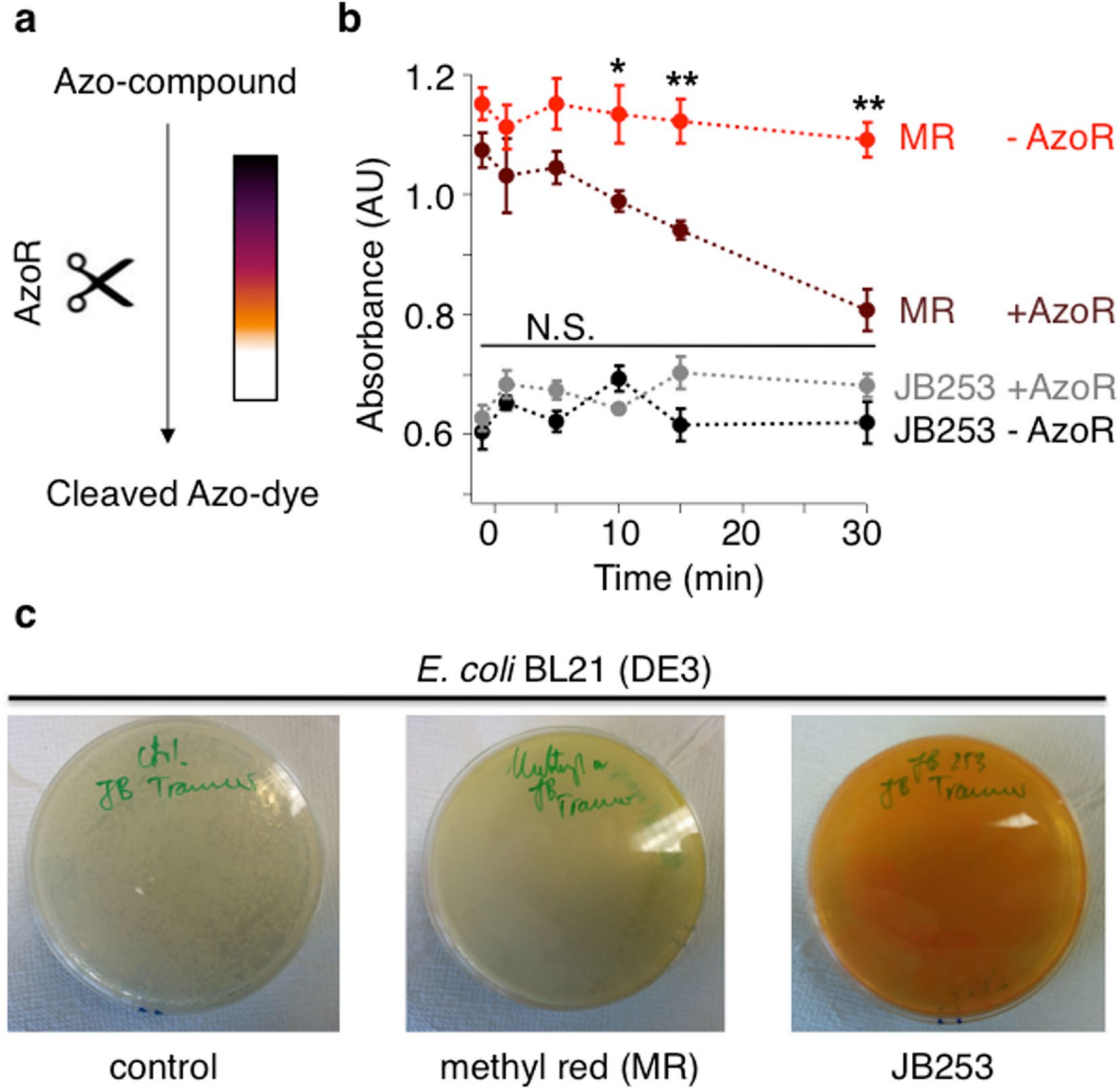
Assessing azo-compound cleavage by azoreductase. (**a**) The bacterial enzyme azoreductase (AzoR) reductively cleaves deeply-colored azo-compounds into corresponding colorless anilines. (**b**) AzoR derived from *E. coli* BL21 (DE3) is able to cleave the azo-dye Methyl Red (MR; λ = 430 nm), but not the AzoSulfonylurea JB253 (λ = 450 nm) (*n* = 4). (**c**) *E. coli* BL21 (DE3) on Agar plates after control (left), MR (middle) and JB253 (right) incubation shows that JB253 is still present while MR almost completely disappears; (*n* = 4). *P < 0.05, **P < 0.01 or NS, non-significant, MR or JB253 − AzoR *versus* MR or JB253 + AzoR; two-way ANOVA (Bonferroni’s multiple comparison test). Values represent mean ± SEM.

Azobenzene moieties can be reductively cleaved by bacteria to form potentially toxic anilines^28^. To test this possibility, assays were performed in the presence of the bacterial enzyme azoreductase (AzoR)^9^, which is found in *E. coli* and *Salmonella* strains normally resident in the gut (Fig. 3a). While AzoR was able to cleave the azo-dye Methyl Red into colorless anilines, JB253 remained unaffected, as assessed using absorbance of the azobenzene band over time (Fig. 3b and c).

### *In vivo* photocontrol of glucose homeostasis

**Figure 4:**
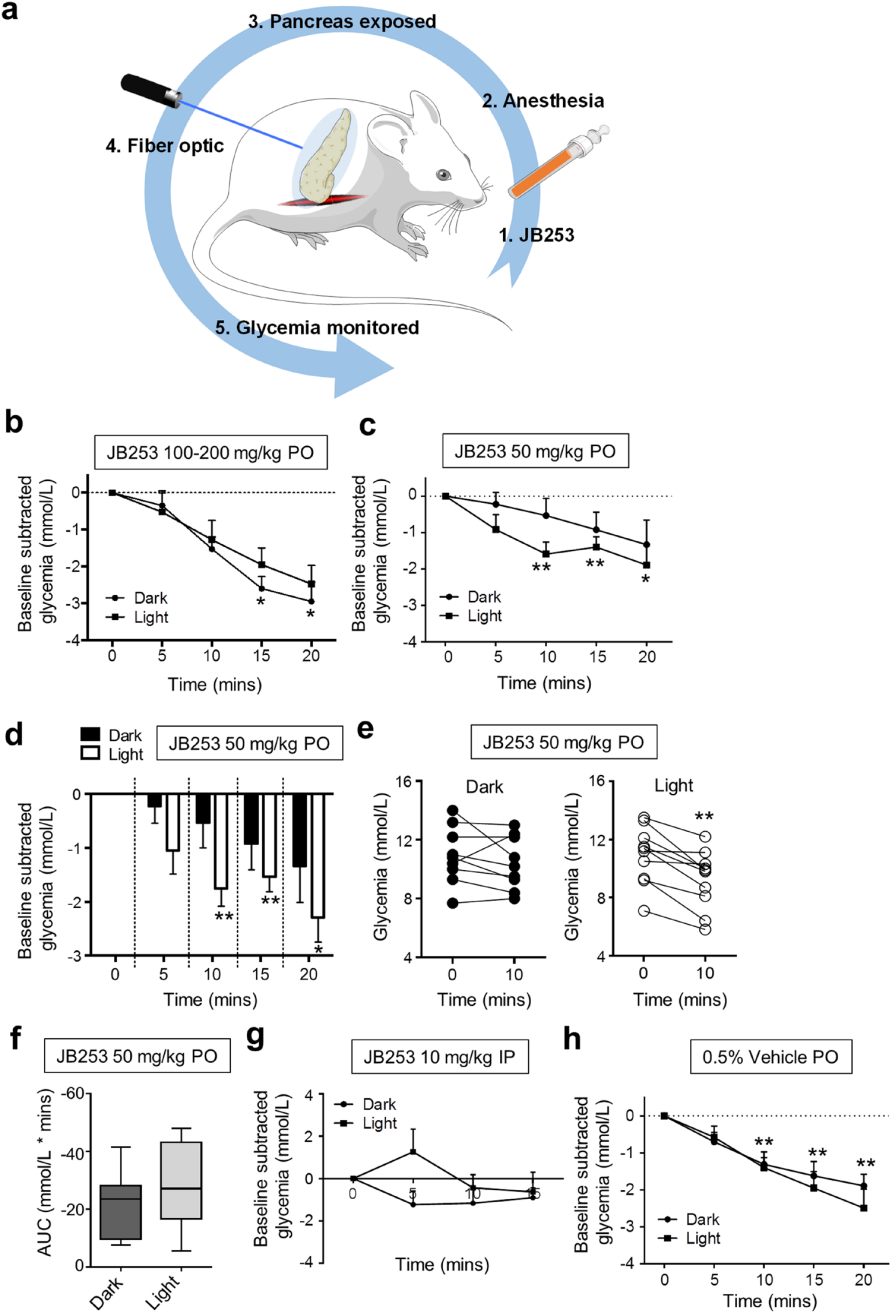
Optical control of glucose homeostasis using JB253. (**a**) Schematic showing the experimental protocol for *in vivo* photopharmacology in mice (adapted from Servier Medical Art under a CC-BY licence) (https://creativecommons.org/licenses/by/3.0/). (**b**) JB253 100–200 mg/kg administered *per os* (PO) significantly decreases glycemia in the dark without evidence of photocontrol following illumination with a λ = 470 ± 5 nm (blue) laser (*n* = 4 animals per state). (**c**) JB253 50 mg/kg administered PO significantly lowers glycemia only during exposure to blue light (λ = 470 nm) (*n* = 9–10 animals per state). (**d**) As for (**c**), but bar graph to better show the differences between light/dark. (**e**) Before-and-after plot showing that all animals receiving JB253 50 mg/kg responded to light with decreases in glucose concentration (*n* = 9–10 animals per state). (**f**) Box and whisker plot showing area-under-the-curve (AUC) in JB253-treated animals under dark or light conditions. (**g**) Light does not significantly influence glycemia in mice injected intraperitoneally with 10 mg/kg JB253 (*n* = 3 animals per state). (**h**) As for (**c**), but showing lack of photocontrol in mice receiving vehicle-alone (0.5% CMC) PO (*n* = 9–10 animals per state). For multiple comparisons, *P < 0.05, **P < 0.01; repeated measures one-way ANOVA (Bonferroni’s posthoc test). For pairwise comparisons (**e** and **f**), **P < 0.01; paired Student’s *t*-test or Mann-Whitney *U* test. Data represent the mean + SEM (or median + range for Box and Whiskers plot).

CD1 mice were orally gavaged with JB253 or vehicle control (0.5% CMC), before general anesthesia with fentanyl/fluanisone. Animals were maintained on a heated stage, anesthesia depth regularly checked using the pedal reflex, and the pancreas continuously irrigated to prevent desiccation. Once stabilized, the splenic portion of the pancreas was exteriorized *via* a left flank laparotomy and a fiber optic ferrule placed in close proximity to the tissue (Fig. 4a). A 50 mW diode-pumped solid-state laser was connected to the fiber input to allow blue light illumination (λ = 470 ± 5 nm) (Fig. 4a).

While high doses (100–200 mg/kg) of JB253 significantly decreased blood glucose levels in the dark, there was no evidence of photocontrol (Fig. 4b). This likely reflects the photostationary state of the compound when interacting with its target, where some K_ATP_ channel block is observed even in the presence of ‘inactive’ (dark) JB253, particularly at high concentrations^10^. To circumvent this, the dose of JB253 was reduced further to decrease the magnitude of resting K_ATP_ channel block. At 50 mg/kg, JB253 in the dark appeared to cause a reduction in glycemia (Fig. 4c). However, no interaction between time and glycemia was detected using ANOVA (P > 0.05). By contrast, illumination of the pancreas with blue light to *cis*-isomerize and activate JB253 significantly lowered glycemia at 10, 15 and 20 min time-points (Fig. 4c and d), and this could not be readily reversed over the short term (Fig. S1). All animals (*n* = 9) receiving JB253 exhibited a decrease in glucose concentration upon exposure to light at 10 min (Fig. 4e). The area-under-the-curve of glycemia tended to be increased in animals receiving both JB253 and illumination (Fig. 4f).

**Figure 5:**
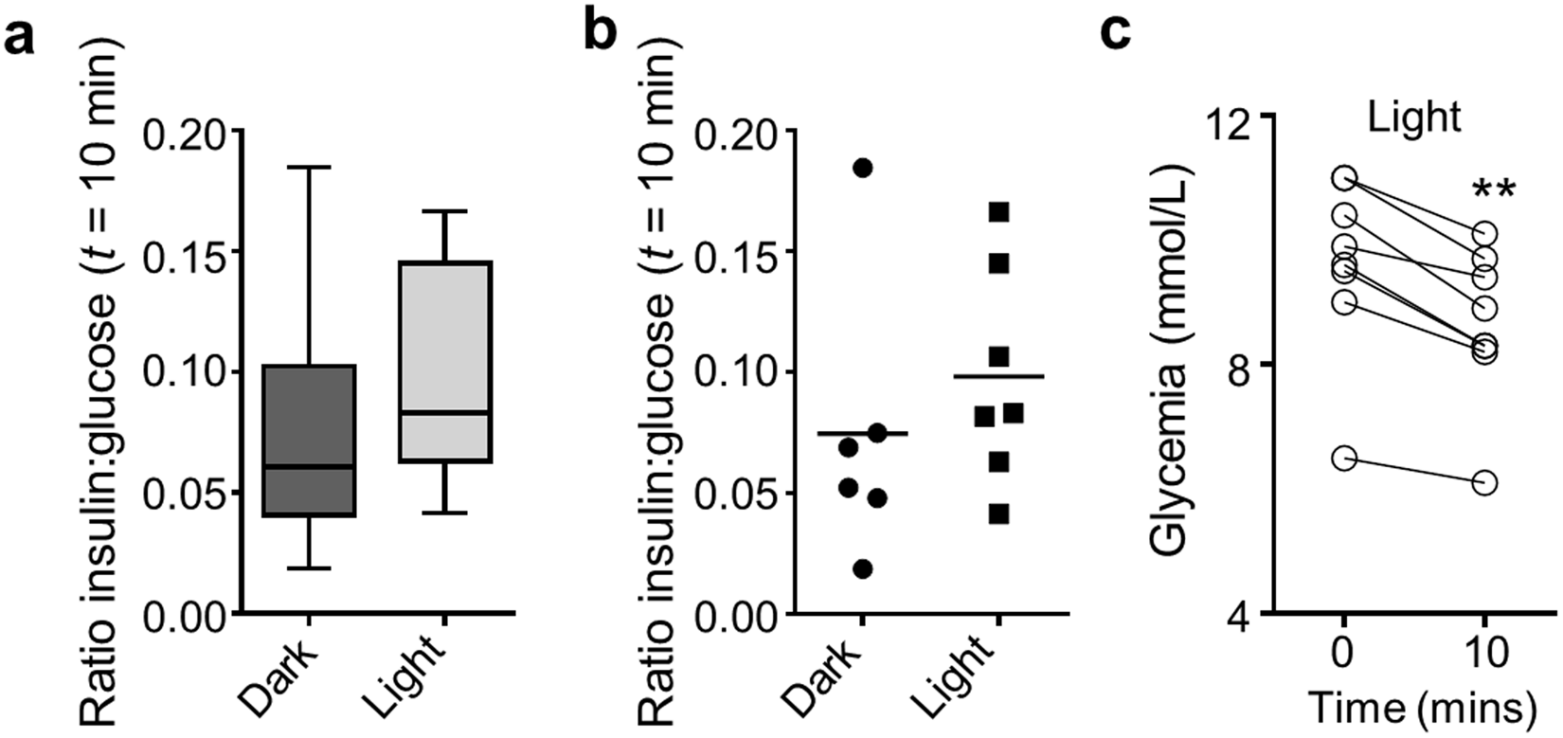
JB253 and insulin secretion. (**a**) Box and whiskers plot showing that insulin secretion, normalized as the ratio insulin:glucose, tends to increase during illumination in JB253-treated animals (median and range shown). (**b**) As for (**a**), but scatter plot to show the presence of a single outlier (mean is shown). (**c**) Before-and-after plot showing that glucose concentrations decreased during illumination in all JB253-treated animals examined. **P < 0.01, paired Student’s *t*-test.

It is unlikely that light alone was able to account for these effects, since intraperitoneal injection of JB253 did not cause a decrease in glucose levels either in the dark or following irradiation with λ = 470 ± 5 nm (Fig. 4g). Moreover, photoswitching was absent in the presence of vehicle (0.5% CMC) alone (Fig. 4h), with almost identical decreases in blood glucose levels under both dark and light conditions. Similar results were seen with DMSO as the vehicle (Fig. S2). Further nonparametric longitudinal analysis using an ANOVA-type statistic showed significant effects of time on glycemia for vehicle and JB253, even in the dark. However, the relative effect size of light on glycemia was much larger for JB253 than vehicle (Supplementary Table 10) (Figs S3 and S4). Lastly, insulin secretion, corrected for glycemia and displayed as the ratio insulin:glucose, tended to increase during light stimulation (Fig. 5). Thus, robust photocontrol of glucose homeostasis was apparent in JB253-treated animals.

## Discussion

The results of the present study demonstrate for the first time the use of photopharmacology to optically control glucose homeostasis *in vivo* in living anesthetized rodents. Alongside this, preliminary toxicology testing revealed no obvious toxicity of the photoswitchable sulfonylurea JB253, even at very high doses administered over 7 days (1000 mg/kg). While some mutagenicity was observed in bacteria, we were unable to detect cleavage into carcinogenic anilines using azoreductase (AzoR) assays.

To enable illumination and activation of JB253, a non-heating fiber optic was placed in close proximity to the splenic portion of the pancreas. Thus, the efficacy of JB253 was likely underestimated, since islets are scattered throughout the pancreas, including a dense population in the gastric portion^29^. While the size of the fiber optic ferrule was limiting in the present study, devices to allow light delivery into discrete body compartments are under rapid development. For example, 3D-printed organ meshes embedded with µLEDs^30^, as well as wireless ‘optofluidic’ devices to deliver drug and light, have recently been described^31^. Moreover, oral delivery of light-emitting capsules might provide another potential mechanism for gastrointestinal illumination.

Mutagenicity studies showed that JB253 is potentially carcinogenic in the presence of S9 in a single strain of *Salmonella* (TA 1537), in addition to *E. coli*. However, the results from the Ames test should be interpreted carefully, since false positives are common^32^. Moreover, a positive Ames test result simply means that a chemical is capable of causing mutagenesis, but does not show whether this leads to cancer or not. Thus, more extensive testing is required in animal models to understand whether JB253 and other azobenzene-bearing compounds such as JB558^9^, LirAzo^19^ and PhotoETP^20^ are in fact carcinogenic.

A potential source of mutagenicity/toxicity is the liberation of reactive anilines following cleavage of the azobenzene by azoreductase resident in gut bacteria^28^. However, we were unable to detect any cleavage following incubation of JB253 with *E. coli* azoreductase. Moreover, no acute toxicity was detected in rats during the repeated dose phase when JB253 was orally administered at 1000 mg/kg for 7 days. Nonetheless, we note that azoreductase effects may be different in the gut, where variants exist, and that such relatively acute treatment may not be sufficient to see any effects from cleavage products. While genotoxicity of lipophilic azo-dyes themselves has been reported^33^, JB253 is polar and water soluble due to its low *pK*_*a*_ = 4.76^10^ and as an anion is therefore unlikely to interact with DNA.

Toxicology testing was performed in rats rather than mice, since the former species is the model preferred by regulatory bodies due to broadly similar pharmacokinetics *versus* humans. Although we did not see any obvious toxicity in mice during 30 min exposure to JB253, suggesting these findings are equally applicable to the latter, we cannot exclude effects over longer time courses.

During control experiments, we noticed a reduction in glycemia over time in anesthetized mice, irrespective of the vehicle used. Anesthesia can induce profound hypoglycemia in small rodents due to their low glycogen reserves^34^ and effects on insulin release^35^, an action likely exacerbated by the long anesthetic durations and young age of the animals used in the present study. An intriguing finding was that JB253 administered intraperitoneally was ineffective. The reasons for this are unknown, but may include poor bioavailability, as postmortem analysis 30 minutes post-injection revealed the presence of residual JB253 in the peritoneal cavity (>80% of the initial administered volume). This could also explain why intraperitoneal injection with either vehicle or JB253 did not reduce glycemia, since they may have been poorly absorbed, or alternatively, evoked a stress response that counteracted the effects of anesthesia. In any case, the administration of sulfonylureas is conventionally *via* the oral route.

JB253 allowed optical control over blood- glucose concentrations, although these actions could not easily be reversed post-illumination. However, it should be noted that: (1) the 20 min experimental time window was the maximum we could safely achieve without lethal hypoglycemia caused by anesthesia plus sulfonylurea treatment; (2) the biological half-life of insulin is ~10 min^36^, meaning that glucose-lowering effects may be present for a short time after cessation of illumination; and (3) sulfonylureas activate non-ionic second messenger pathways (*e.g.* Epac2^37, 38^) that may display persistent signaling. Thus, we cannot exclude the reversible optical control of blood- glucose using the present paradigm. Along similar lines, JB253 only tended to increase insulin secretion during illumination. However, further studies are warranted with larger cohorts given the variability inherent to this approach/presence of outliers, as well as stimulatory effects of anesthesia on beta cell function.

JB253 binds to both SUR1 and Epac2 to close K_ATP_ channels *in vitro* ^9, 10^. Whether similar mechanisms account for the *in vivo* effects of JB253 is unknown. Therefore, further studies using double *SUR1* ^*−*/*−*^ *Epac2* ^*−*/*−*^ knockout animals^38, 39^ are warranted in the future. While the *in vivo* pharmacokinetics/pharmacodynamics of JB253 were not assessed, previous crystallographic studies have shown a high degree of three-dimensional structural similarity with glimepiride^10^, which was clearly effective at lowering blood glucose in mice in the present studies. Furthermore, glucose monitoring was always commenced within 15–30 min of dosage, meaning that the drug was unlikely to be eliminated even if its half-life was drastically reduced in rodents.

In summary, JB253 allows the remote control of glucose homeostasis in rodents with a reasonable safety margin. Together with advances in bioengineering, this may open up the possibility to one day deploy photopharmacology for the treatment of T2D in humans. Importantly, photopharmacology has the potential to re-purpose well-characterized and inexpensive drugs such as the sulfonylureas, while at the same time reducing toxicity associated with off-target effects or prolonged activity profiles. More broadly, these studies are applicable to other photopharmacological agents including those intended to treat blindness^13^, infection^14, 15^ and cancer^16, 17^.

## Methods

### Study approval

Experimental protocols were approved by Imperial College London’s Animal Welfare and Ethical Review Body (AWERB) and carried out in accordance with the Animals (Scientific Procedures) Act 1986 of the United Kingdom under Home Office PPL 70/7349 (“Regulation of Glucose Homeostasis *In Vivo*”) and PPL 60/4185 (“Toxicology of Pharmaceuticals”).

### JB253 bulk synthesis

Sulfanilamide (10.0 g, 58.1 mmol, 1.0 equiv.) was dissolved in 2.4 m HCl (300 mL) and cooled to 0 °C. Under vigorous stirring, a solution of 2.3 m NaNO_2_ (30 mL) was added drop-wise until the mixture turned homogeneous and turned pale yellow. The formed diazonium salt was stirred under ice-cooling for an additional 10 min before it was transferred drop-wise into a solution of *N,N*-diethylaniline (8.65 g, 58.1 mmol, 9.3 mL, 1.0 equiv.) in 1 m NaOAc (200 mL) and MeOH (20 mL). The solution turned to dark red and was allowed to warm to room temperature under stirring. The crude product was extracted with EtOAc (3x), and the combined organic layers were washed with brine and dried over Na_2_SO_4_, filtered and evaporated to obtain 18.4 g of crude (*E*)-4-((4-(diethylamino)phenyl)diazenyl)benzenesulfonamide as a red solid.

A 1 L round-bottom flask was charged with 18.4 g of crude (*E*)-4-((4-(diethylamino)phenyl)diazenyl)benzenesulfonamide and K_2_CO_3_ (8.0 g, 58.1 mmol, 1.0 equiv.) in acetone (400 mL). The reaction mixture was heated to 80 °C for 30 min and cyclohexyl isocyanate (7.27 g, 58.0 mmol, 7.4 mL, 1.0 equiv.) was added drop-wise into the hot solution. The reaction temperature was maintained at 80 °C for 5 h and a precipitate formed, which was collected after cooling to room temperature by means of filtration. The solid was taken up in EtOAc (500 mL) and washed successively with 1 m HCl (2 × 250 mL) and brine (500 mL). The organic layer was heated to 100 °C and the hot suspension filtered to remove impurities. The filtrate was allowed to crystallize over night at 4 °C to give JB253 as a crystalline red solid that was collected by filtration. The mother liquor could be concentrated and treated the same way as described above to maximize yield up to 59%, yielding 15.8 g (34.5 mmol) of JB253 over two steps. JB253 crystals were co-evaporated from DCM (2 × 100 mL) to remove residual EtOAc and finally dried under HV conditions. While LCMS data confirmed the high purity of JB253 (Fig. S5), the ^1^H NMR spectroscopy did not exactly match that acquired previously^10^. Although all expected signals were present (Supplementary Table 7), accounting for the high purity of JB253, they were shifted slightly downfield. We thought that this may be a pH issue and therefore titrated the JB253 solution in DMSO-*d*_6_ with NaOD. Indeed, increasing base shifted the aromatic and aliphatic signals upfield (Supplementary Table 8), confirming that the desired product was obtained (*cf* mass spectrometry; Supplementary Table 9) and signals are affected by the protic environment of the sample.

### Mutagenicity and toxicology testing

JB253 was tested for mutagenic activity in strains *Salmonella typhimurium* TA 1535, TA 100, TA 1537, TA 98 and *Escherichia coli* WP2uvrA. The mutation test was conducted by the Direct Plate Incorporation Method on agar plates in triplicate in the absence and presence of an Aroclor 1254-induced rat liver S9 preparation and the co-factors required for mixed-function oxidase activity (S9 mix). JB253 was dosed at 7 concentrations, ranging from 5 to 5000 µg per plate in the absence and presence of S9 mix.

JB253 was tested at escalating dose levels in Han Wistar rats to determine a maximum tolerated dose (MTD), followed by a repeat dose phase (RDP) for 7 days (see Supplementary Tables 5 and 6 for details of experimental groups). The test item was administered in 0.5% w/v carboxymethylcellulose (CMC; medium viscosity) in water. Throughout, body weight and food consumption were monitored and clinical signs and gross necropsy findings reported. In all cases, studies were conducted on-site at Charles River Laboratories UK and performed in accordance with the OECD Principles of Good Laboratory Practice, as incorporated into the United Kingdom Statutory Instrument for GLP and as accepted by Regulatory Authorities throughout the European Union, United States of America (FDA and EPA) and Japan (MHLW, MAFF and METI) and other countries that are signatories to the OECD Mutual Acceptance of Data Agreement. All procedures and animal numbers were the maximum allowed under UK Home Office regulations concerning toxicology testing, and deemed adequate for submission by the above regulatory bodies.

### Azoreductase assay

The reductive cleavage of an azobenzene diazene bond into the corresponding anilines was assessed as previously described^9^. Briefly, *Escherichia coli* (BL21 DE3) cells were transformed with pcDNA3.1 and outgrown in LB media with ampicillin (5 mL) to an OD_600_ = 0.6. Methyl red (MR) or JB253 was added from a 50 mm stock solution in DMSO to a final concentration of 50 μm with or without bacteria. DMSO-alone served as a control. Solutions were inoculated at 37 °C and 180 rpm and aliquots (200 μL) were taken 1 min before (−1 min) and 1, 5, 10, 15 and 30 min after addition. The bacteria-containing tubes were centrifuged for 1 min (4 °C and 12,000 rpm) and then stored immediately on ice for the duration of the experiment. The supernatant (100 µL) of each Eppendorf tube was transferred into a well of a 96-well plate (clear, flat bottom, Greiner) and absorbance measured at the respective λ_max_ (MR: 430 nm; JB253: 450 nm) with a BMG plate reader. In parallel, 250 µL of each sample was plated on an Agar plate containing ampicillin and incubated at 37 °C overnight for 16 h. Pictures were taken to show bacterial growth and the disappearance of MR but not JB253. DMSO-alone served as control.

### Islet isolation

CD1 mice (8–12 weeks) were euthanized by cervical dislocation, the bile duct clamped at the duodenal ampulla, and collagenase solution (1 mg/mL) injected. Islets were isolated using a Histopaque gradient and cultured overnight in solution containing Roswell Park Memorial Institute (RPMI) medium supplemented with 10% fetal calf serum, 100 U/mL penicillin and 100 µg/mL streptomycin.

### Calcium imaging

Islets were loaded with the Ca^2+^ indicator Fluo8 before imaging using a Crest X-Light spinning disk head coupled to a Nikon Ti-E microscope base and a 10x/0.4NA objective. Pulsed illumination (280 ms) was delivered every 5 s at λ = 458–482 nm using a Lumencor Spectra X light engine. Activation of JB253 was achieved by switching to constant illumination at λ = 458–482 nm. Emitted signals were detected at λ = 500–550 nm using a Photometrics Evolve Delta 512 EMCCD. In all cases, HEPES-bicarbonate buffer was used, containing in mm: 120 NaCl, 4.8 KCl, 24 NaHCO_3_, 0.5 Na_2_HPO_4_, 5 HEPES, 2.5 CaCl_2_, 1.2 MgCl_2_, 8 *D*-glucose. Treatments were applied at the indicated times and concentrations. Ca^2+^ traces were normalized as F/F_min_ (F = fluorescence at any given timepoint; F_min_ = minimum fluorescence) and the relative change in this value over time denoted by a scale bar.

### Histopathology

Hearts were fixed in 4% (v/v) formalin and wax-embedded. Coronal sections were cut at 5 µm on a microtome, mounted on Superfrost slides, wax removed using Histoclear, and tissue rehydrated by passing through serial dilutions of industrial denatured alcohol and distilled water. Counterstaining was performed using hematoxylin and eosin before capturing images using a Leica DM IL light microscope.

### *In vivo* studies

Female CD1 mice (8–12 weeks; ~30 g) were maintained in a specific pathogen-free (SPF) facility under a 12 h light–dark cycle with *ad libitum* access to water and standard laboratory diet. Animals were gavaged *per os* with the indicated drug dissolved in 0.5% CMC in water, before general anesthesia using intraperitoneal injection of 10 µL/g fentanyl/fluanisone (Hypnorm). Following injection of the local anesthetic bupivacaine into the incision site, left flank laparotomy was performed under aseptic conditions to expose the spleen, and a fiber optic placed in proximity to the pancreas (1.25 mm diameter). Glucose concentration was measured *via* lateral tail vein incision at 0, 5, 10, 15 and 20 min post-surgery with and without illumination using a λ = 470 ± 5 nm laser (Crystalaser). Since we were unable to fast animals before surgery, baseline glycemia was variable and therefore blood-glucose at *t* = 0 min was subtracted from each subsequent time point to give the ‘baseline subtracted change in glycemia (mmol/L)’. It should be further noted that glycemia tended to reduce during anesthesia, most likely due to depletion of glucose reserves^34^. Glucose monitoring was commenced 5–15 min following oral gavage with vehicle or drug. Plasma insulin concentrations at *t* = 10 min were measured following tail vein bleed using a Cisbio insulin mouse serum assay kit (http://www.cisbio.com/usa/drug-discovery/insulin-mouse-serum-assay-kit) according to the manufacturer’s instruction. Throughout, animals were maintained at 38 °C using a heated stage and the pancreas continuously irrigated with saline solution (0.9% NaCl) to avoid desiccation. Mice rather than rats were used for these studies, since they possess a smaller pancreas, which is more amenable to illumination with a fiber optic. Moreover, *in vitro* studies were performed in mouse tissue, and the majority of JB253 provided for research purposes so far has been used in this species.

### Statistics

Two-way ANOVA did not show a significant interaction between illumination and glycemia for JB253 (P = 0.58) and vehicle-treated (P = 0.53) animals. Therefore, light and dark datasets for all treatments were considered independently using either: (1) repeated measures one-way ANOVA followed by Bonferroni’s post hoc test; or (2) nonparametric analysis of longitudinal data in factorial experiments^40^. When only two groups were considered, comparisons were made using Student’s paired *t*-test or Mann-Whitey *U* test. Analyses were performed using Graphpad Prism 6.0 (Graphpad Software) or R-Project (“nparLD” package) and results deemed significant at P < 0.05. Unless otherwise stated, data are presented as the mean ± SEM.

## Electronic supplementary material

Supplementary Information
