# Supplementary material for: Remote control of glucose homeostasis in vivo using photopharmacology
Source: Sci Rep. 2017 Mar 22;7:291. doi: 10.1038/s41598-017-00397-0 (PMC5428208; doi:10.1038/s41598-017-00397-0)
Supplement: Supplementary file 1 — Supplementary Information [file 41598_2017_397_MOESM1_ESM.doc]

**Supplementary INFORMATION**

**Remote control of glucose homeostasis *in vivo* using photopharmacology**

Zenobia B. Mehta^1,6^, Natalie R. Johnston^1,6^, Marie-Sophie Nguyen-Tu^1,6^, Johannes Broichhagen^2,5^, Peter Schultz^2^, Dean P. Larner^3,4^, Isabelle Leclerc^1^, Dirk Trauner*^2^, Guy A. Rutter*^1^ and David J. Hodson*^3,4^

^1^Section of Cell Biology and Functional Genomics, Department of Medicine, Imperial College London, W12 0NN, UK. ^2^Department of Chemistry and Center for Integrated Protein Science, LMU Munich, Munich, Germany. ^3^Institute of Metabolism and Systems Research (IMSR) and Centre of Membrane Proteins and Receptors (COMPARE), University of Birmingham, Edgbaston, B15 2TT, UK. ^4^Centre for Endocrinology, Diabetes and Metabolism (CEDAM), Birmingham Health Partners, Birmingham, B15 2TH, UK.

^5^Present address: Max-Planck Institute for Medical Research, Jahnstr. 29, 69120 Heidelberg, Germany.

^6^These authors contributed equally.

*Correspondence

[d.hodson@bham.ac.uk](mailto:d.hodson@bham.ac.uk)

[d.trauner@lmu.de](mailto:d.trauner@lmu.de)

[g.rutter@imperial.ac.uk](mailto:g.rutter@imperial.ac.uk)

**General**

NMR spectra were recorded in deuterated DMSO on a BRUKER Avance III HD 400 (equipped with a CryoProbeTM) instrument and calibrated to residual solvent peaks (^1^H in ppm): DMSO-*d*_6_ (2.50). Multiplicities are abbreviated as follows: s = singlet, d = doublet, t = triplet, q = quartet, p = pentet, br = broad, m = multiplet. Spectra are reported based on appearance, not on theoretical multiplicities derived from structural information.

LCMS was performed on an Agilent 1260 Infinity HPLC System, MS-Agilent 1100 Series, Type: 1946D, Model: SL, equipped with a Agilent Zorbax Eclipse Plus C18 (100 x 4.6 mm, particle size 3.5 micron) RP column with a constant flow-rate of 1 mL/min. A gradient over 10 minutes was used as follows: MeCN/H_2_O/formic acid = 10/90/0.1 🡪 90/10/0.1 (8 min) 🡪 100/0/0.1 (10 min).

**Supplementary Table 1:** Mutation assay in *Salmonella* TA 1535, TA 1537, TA 98 and TA 100 without metabolic activation.

| Strain | Test item | Dose level per plate | Mean revertants per plate | Standard Deviation | Ratio treated / solvent | Individual revertant  colony counts |
| --- | --- | --- | --- | --- | --- | --- |

|  |  |  |  |  |  |  |
| --- | --- | --- | --- | --- | --- | --- |
| **TA 1535** | **JB253** | 5 µg | *8.3* | *3.2* | *1.0* | 6, 7, 12 |
|  |  | 17 µg | *7.7* | *3.2* | *1.0* | 4, 10, 9 HI |
|  |  | 50 µg | *7.7* | *3.1* | *1.0* | 7, 5, 11 |
|  |  | 167 µg | *8.3* | *3.2* | *1.0* | 7 P, 6 P, 12 P |
|  |  | 500 µg | *6.0* | *1.0* | *0.8* | 5 HIP, 7 P, 6 P |
|  |  | 1667 µg | *7.0* | *2.0* | *0.9* | 9 P HP, 5 P HP, 7 P HP |
|  |  | 5000 µg | *5.0* | *2.0* | *0.6* | 5 P HP, 3 P HP, 7 P HP |
|  | **DMSO** | - | *8.0* | *2.6* | *-* | 7, 11, 6 |
|  |  |  |  |  |  |  |

|  |  |  |  |  |  |  |
| --- | --- | --- | --- | --- | --- | --- |
| **TA 1537** | **JB253** | 5 µg | *13.3* | *2.3* | *1.7* | 12, 12, 16 |
|  |  | 17 µg | *11.0* | *5.3* | *1.4* | 17, 9, 7 |
|  |  | 50 µg | *4.0* | *2.0* | *0.5* | 2, 6, 4 |
|  |  | 167 µg | *10.3* | *5.1* | *1.3* | 9 P, 16 P, 6 P |
|  |  | 500 µg | *16.3* | *4.5* | *2.0* | 12 P, 16 P, 21 P |
|  |  | 1667 µg | *10.0* | *4.0* | *1.3* | 10 P HP, 14 P HP, 6 P HP |
|  |  | 5000 µg | *7.0* | *1.0* | *0.9* | 7 P HP, 6 P HP, 8 P HP |
|  | **DMSO** | - | *8.0* | *3.5* | *-* | 10, 10, 4 |
|  |  |  |  |  |  |  |

|  |  |  |  |  |  |  |
| --- | --- | --- | --- | --- | --- | --- |
| **TA 98** | **JB253** | 5 µg | *18.3* | *6.4* | *0.8* | 11, 23, 21 |
|  |  | 17 µg | *17.0* | *3.6* | *0.8* | 18, 20, 13 |
|  |  | 50 µg | *19.0* | *15.1* | *0.9* | 5, 35, 17 |
|  |  | 167 µg | *30.0* | *19.7* | *1.4* | 48 P, 9 P, 33 P |
|  |  | 500 µg | *23.3* | *10.3* | *1.1* | 26 P, 32 P, 12 P |
|  |  | 1667 µg | *20.7* | *8.7* | *1.0* | 23 HPP, 11 HPP, 28 HPP |
|  |  | 5000 µg | *22.0* | *6.2* | *1.0* | 20 HPP, 17 HPP, 29 HPP |
|  | **DMSO** | - | *21.7* | *4.7* | *-* | 18, 20, 27 |
|  |  |  |  |  |  |  |

|  |  |  |  |  |  |  |
| --- | --- | --- | --- | --- | --- | --- |
| **TA 100** | **JB253** | 5 µg | *80.3* | *11.7* | *0.9* | 93, 78, 70 |
|  |  | 17 µg | *78.7* | *4.7* | *0.9* | 75, 84, 77 |
|  |  | 50 µg | *78.7* | *8.0* | *0.9* | 71, 78, 87 |
|  |  | 167 µg | *76.3* | *2.3* | *0.9* | 79 P, 75 P, 75 P |
|  |  | 500 µg | *104.7* | *13.8* | *1.2* | 115 P, 110 P, 89 P |
|  |  | 1667 µg | *90.7* | *21.4* | *1.1* | 72 P, 114 P, 86 P |
|  |  | 5000 µg | *67.7* | *8.3* | *0.8* | 65 P HP, 77 P HP, 61 P HP |
|  | **DMSO** | - | *86.3* | *12.5* | *-* | 92, 72, 95 |
|  |  |  |  |  |  |  |

|  | | Key to Plate Postfix Codes | |
| --- | --- | --- | --- |
|  |  |  |  |
|  |  | P  HP  HI | Precipitate  Hand Counted Due to Precipitation  Hand Counted Due to Interference |

**Supplementary Table 2:** Mutation assay in *E. Coli* WP2uvrA, and positive controls in *E. Coli* WP2uvrA and *Salmonella* TA 1535, TA 1537, TA 98 and TA 100 without metabolic activation.

| Strain | Test item | Dose level per plate | Mean revertants per plate | Standard Deviation | Ratio treated / solvent | Individual revertant  colony counts |
| --- | --- | --- | --- | --- | --- | --- |

|  |  |  |  |  |  |  |
| --- | --- | --- | --- | --- | --- | --- |
| **WP2uvrA** | **JB253** | 5 µg | *10.7* | *4.5* | *0.9* | 15, 6, 11 |
|  |  | 17 µg | *10.3* | *1.2* | *0.9* | 11, 9, 11 HI |
|  |  | 50 µg | *9.0* | *2.0* | *0.8* | 9, 7, 11 |
|  |  | 167 µg | *6.3* | *3.2* | *0.5* | 10 P, 4 P, 5 P |
|  |  | 500 µg | *15.7* | *2.5* | *1.3* | 18 P, 16 P, 13 P |
|  |  | 1667 µg | *7.7* | *1.2* | *0.6* | 7 P HP, 9 P HP, 7 P HP |
|  |  | 5000 µg | *10.3* | *4.5* | *0.9* | 10 P HP, 6 P HP, 15 P HP |
|  | **DMSO** | - | *12.0* | *3.6* | *-* | 16, 11, 9 |
|  |  |  |  |  |  |  |

|  |  |  |  |  |  |  |
| --- | --- | --- | --- | --- | --- | --- |
| **TA 1535** | **NaN_3_** | 1 µg | *254.0* | *33.0* | *31.8* | 238, 232, 292 |
| **TA 1537** | **9AA** | 80 µg | *1887.3* | *407.9* | *235.9* | 1426, 2036, 2200 |
| **TA 98** | **2NF** | 1 µg | *272.3* | *7.1* | *12.6* | 271, 266, 280 |
| **TA 100** | **NaN_3_** | 1 µg | *781.3* | *65.7* | *9.1* | 855, 729, 760 |
| **WP2uvrA** | **ENNG** | 2 µg | *147.7* | *60.2* | *12.3* | 83, 202, 158 |
|  |  |  |  |  |  |  |

| Key to Positive Controls | | Key to Plate Postfix Codes | |
| --- | --- | --- | --- |
|  |  |  |  |
| NaN_3_  9AA  2NF  ENNG | Sodium Azide  9-Aminoacridine  2-Nitrofluorene  N-Ethyl-N-Nitro-N-Nitrosoguanidine | P  HP  HI | Precipitate  Hand Counted Due to Precipitation  Hand Counted Due to Interference |

**Supplementary Table 3:** Mutation assay in *Salmonella* TA 1535, TA 1537, TA 98 and TA 100 with metabolic activation.

| Strain | Test item | Dose level per plate | Mean revertants per plate | Standard Deviation | Ratio treated / solvent | Individual revertant  colony counts |
| --- | --- | --- | --- | --- | --- | --- |

|  |  |  |  |  |  |  |
| --- | --- | --- | --- | --- | --- | --- |
| **TA 1535** | **JB253** | 5 µg | *11.7* | *6.0* | *2.5* | 6, 18, 11 |
|  |  | 17 µg | *9.0* | *3.5* | *1.9* | 11, 5, 11 |
|  |  | 50 µg | *12.3* | *1.2* | *2.6* | 11, 13, 13 |
|  |  | 167 µg | *8.0* | *3.6* | *1.7* | 5 P, 7 P, 12 P |
|  |  | 500 µg | *5.3* | *4.0* | *1.1* | 9 P, 1 P, 6 P |
|  |  | 1667 µg | *11.0* | *2.6* | *2.4* | 13 P, 12 P, 8 HIP |
|  |  | 5000 µg | *8.3* | *1.5* | *1.8* | 8 HIP TL, 10 P TL, 7 P TL |
|  | **DMSO** | - | *4.7* | *3.2* | *-* | 6 HI, 1, 7 HI |
|  |  |  |  |  |  |  |

|  |  |  |  |  |  |  |
| --- | --- | --- | --- | --- | --- | --- |
| **TA 1537** | **JB253** | 5 µg | *12.7* | *2.1* | *1.7* | 15, 11, 12 |
|  |  | 17 µg | *25.7* | *2.5* | *3.3* | 28, 23, 26 |
|  |  | 50 µg | *24.7* | *3.2* | *3.2* | 21, 26, 27 |
|  |  | 167 µg | *10.7* | *3.2* | *1.4* | 7, 13, 12 |
|  |  | 500 µg | *18.3* | *6.8* | *2.4* | 13 P, 16 P, 26 P |
|  |  | 1667 µg | *15.7* | *2.5* | *2.0* | 13 P, 16 P, 18 P |
|  |  | 5000 µg | *16.0* | *11.5* | *2.1* | 29 P TL, 12 P TL, 7 P TL |
|  | **DMSO** | - | *7.7* | *2.3* | *-* | 9, 9 HI, 5 |
|  |  |  |  |  |  |  |

|  |  |  |  |  |  |  |
| --- | --- | --- | --- | --- | --- | --- |
| **TA 98** | **JB253** | 5 µg | *28.3* | *6.4* | *0.8* | 32, 21, 32 |
|  |  | 17 µg | *31.7* | *2.5* | *0.9* | 32, 29, 34 |
|  |  | 50 µg | *25.7* | *8.1* | *0.7* | 21, 35, 21 |
|  |  | 167 µg | *30.3* | *8.6* | *0.9* | 38 P, 32 P, 21 P |
|  |  | 500 µg | *33.0* | *4.0* | *0.9* | 29 P, 33 P, 37 P |
|  |  | 1667 µg | *31.3* | *5.9* | *0.9* | 29 P, 38 P, 27 P |
|  |  | 5000 µg | *36.7* | *10.1* | *1.0* | 46 P TL, 26 P TL, 38 P TL |
|  | **DMSO** | - | *35.0* | *8.5* | *-* | 34, 27, 44 |
|  |  |  |  |  |  |  |

|  |  |  |  |  |  |  |
| --- | --- | --- | --- | --- | --- | --- |
| **TA 100** | **JB253** | 5 µg | *80.0* | *3.6* | *0.9* | 84, 77, 79 |
|  |  | 17 µg | *93.0* | *11.8* | *1.0* | 106, 90, 83 |
|  |  | 50 µg | *84.7* | *12.4* | *1.0* | 77, 99, 78 |
|  |  | 167 µg | *92.7* | *15.5* | *1.0* | 77 P, 93 P, 108 P |
|  |  | 500 µg | *94.0* | *13.1* | *1.1* | 108 P, 92 P, 82 P |
|  |  | 1667 µg | *66.0* | *10.0* | *0.7* | 56 P, 66 P, 76 P |
|  |  | 5000 µg | *84.3* | *8.1* | *0.9* | 75 P, 88 P, 90 P |
|  | **DMSO** | - | *89.0* | *4.4* | *-* | 87, 86, 94 |
|  |  |  |  |  |  |  |

|  | | Key to Plate Postfix Codes | |
| --- | --- | --- | --- |
|  |  |  |  |
|  |  | P  HI  TL | Precipitate  Hand Counted Due to Interference  Thin Lawn |

**Supplementary Table 4:** Mutation assay in *E. Coli* WP2uvrA, and positive controls in *E. Coli* WP2uvrA and *Salmonella* TA 1535, TA 1537, TA 98 and TA 100 with metabolic activation.

| Strain | Test item | Dose level per plate | Mean revertants per plate | Standard Deviation | Ratio treated / solvent | Individual revertant  colony counts |
| --- | --- | --- | --- | --- | --- | --- |

|  |  |  |  |  |  |  |
| --- | --- | --- | --- | --- | --- | --- |
| **WP2uvrA** | **JB253** | 5 µg | *15.0* | *3.0* | *1.6* | 12, 18, 15 |
|  |  | 17 µg | *15.3* | *2.1* | *1.6* | 13, 16, 17 |
|  |  | 50 µg | *14.0* | *3.6* | *1.4* | 10, 17, 15 |
|  |  | 167 µg | *17.3* | *2.5* | *1.8* | 15 HIP, 17 P, 20 P |
|  |  | 500 µg | *21.7* | *1.2* | *2.2* | 21 P, 21 P, 23 P |
|  |  | 1667 µg | *31.0* | *9.5* | *3.2* | 21 P, 32 P, 40 P |
|  |  | 5000 µg | *34.0* | *7.2* | *3.5* | 26 P, 36 HP, 40 HP |
|  | **DMSO** | - | *9.7* | *2.5* | *-* | 10, 7, 12 |
|  |  |  |  |  |  |  |

|  |  |  |  |  |  |  |
| --- | --- | --- | --- | --- | --- | --- |
| **TA 1535** | **2AAN** | 2 µg | *172.0* | *11.5* | *36.9* | 159, 181, 176 |
| **TA 1537** | **2AAN** | 2 µg | *153.0* | *21.9* | *20.0* | 177, 134, 148 |
| **TA 98** | **2AAN** | 0.5 µg | *410.0* | *92.1* | *11.7* | 404, 505, 321 |
| **TA 100** | **2AAN** | 0.5 µg | *553.0* | *24.5* | *6.2* | 578, 552, 529 |
| **WP2uvrA** | **2AAN** | 20 µg | *442.3* | *23.5* | *45.8* | 419, 442, 466 |
|  |  |  |  |  |  |  |

| Key to Positive Controls | | Key to Plate Postfix Codes | |
| --- | --- | --- | --- |
|  |  |  |  |
| 2AAN | 2-Aminoanthracene | HI  P  HP | Hand Counted Due to Interference  Precipitate  Hand Counted Due to Precipitation |

**Supplementary Table 5:** Maximum tolerated dose (MTD) phase for 3 consecutive days

| **Group No.** | **Animal ID** | **Dosage Level**  **(mg/kg)** | **Dosage Concentration**  **(mg/mL)** | **Dosage Volume**  **(mL/kg)** |
| --- | --- | --- | --- | --- |
| 1 | 1001 | 100 | 10 | 10 |
| 2 | 2001 | 300 | 30 | 10 |
| 3 | 3001 | 1000 | 100 | 10 |

**Supplementary Table 6:** 7-day Repeat Dose Phase

| **Group No.** | **Animal ID** | **Dosage Level**  **(mg/kg)** | **Dosage Concentration**  **(mg/mL)** | **Dosage Volume**  **(mL/kg)** |
| --- | --- | --- | --- | --- |
| 4 | 4001-4005 | 1000 | 100 | 10 |

**Supplementary Table 7:** ^1^H NMR (400 MHz, DMSO-*d*_6_) δ

| JB253 | 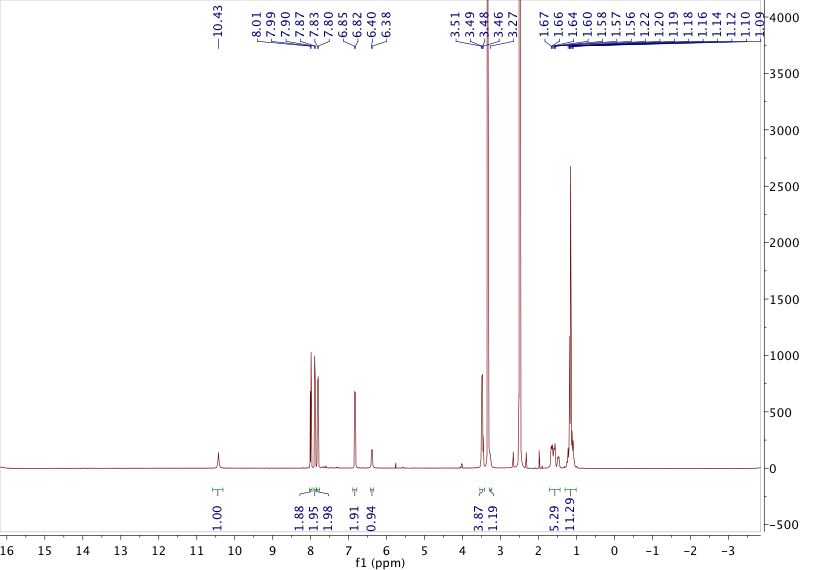10.43 (s, 1H), 8.00 (d, *J* = 8.7 Hz, 2H), 7.89 (d, *J* = 8.7 Hz, 2H), 7.81 (d, *J* = 9.2 Hz, 2H), 6.83 (d, *J* = 9.3 Hz, 2H), 6.39 (d, *J* = 7.9 Hz, 2H), 3.49 (q, *J* = 7.0 Hz, 4H), 3.27 (s, 1H), 1.71–1.42 (m, 5H), 1.23–1.09 (m, 11H). |
| --- | --- |
| JB253 +  15 µM NaOD | 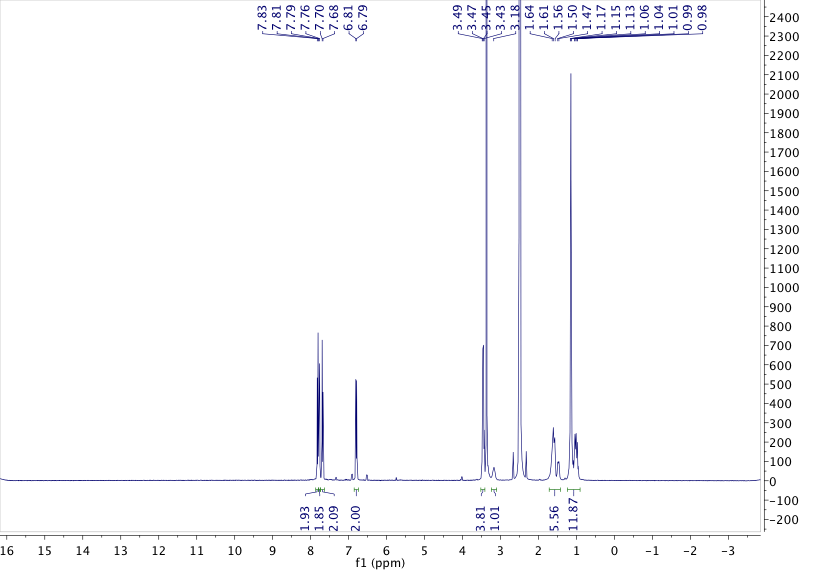  7.82 (d, *J* = 8.5 Hz, 2H), 7.78 (d, *J* = 9.1 Hz, 2H), 7.69 (d, *J* = 8.5 Hz, 2H), 6.80 (d, *J* = 9.2 Hz, 2H), 3.46 (q, *J* = 7.0 Hz, 4H), 3.18 (s, 1H), 1.70–1.43 (m, 5H), 1.20–0.94 (m, 11H). |

**Supplementary Table 8:** comparison of ^1^H NMR of JB253 (red) and JB253 + 15 µM NaOD (blue)

| 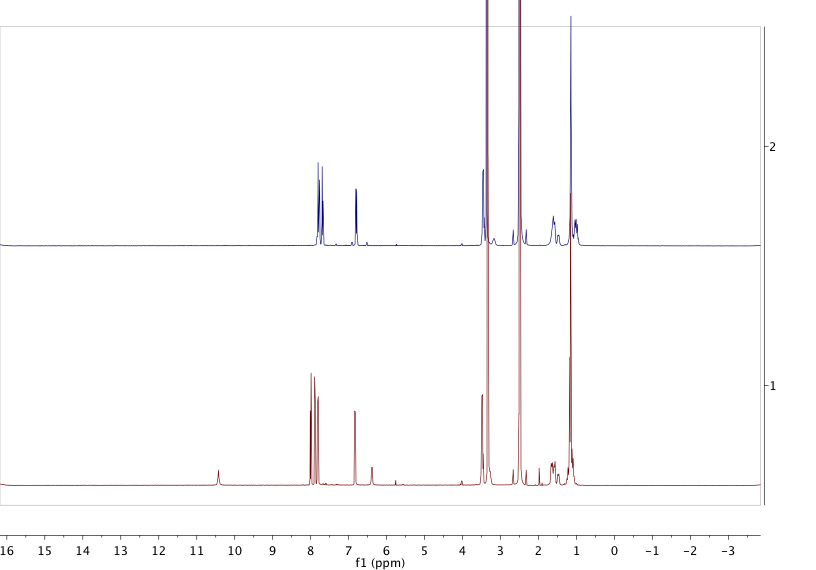 |
| --- |
| ~~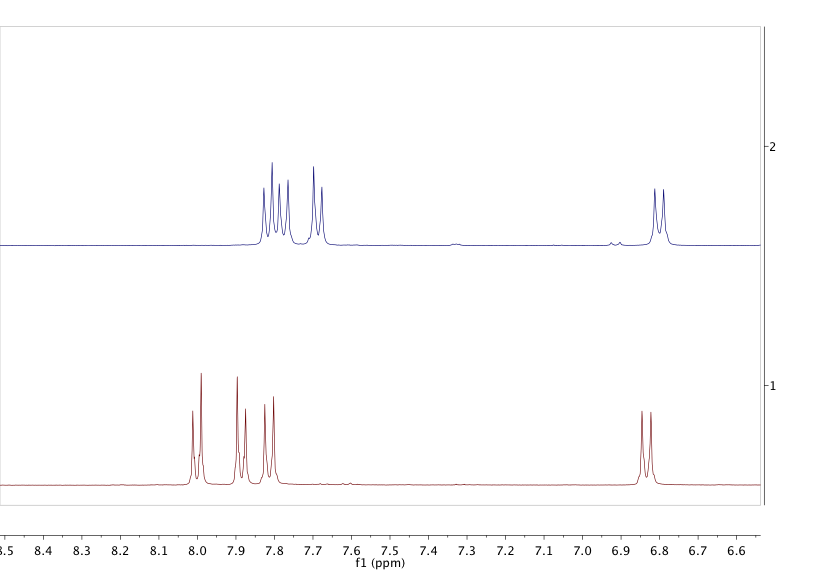~~ |
| ~~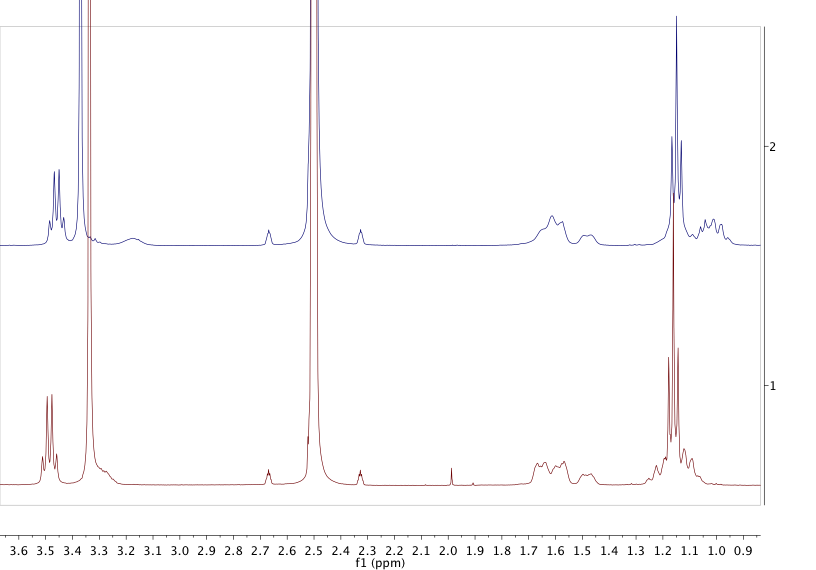~~ |

**Supplementary Table 9:** LRMS of JB253

| LRMS (ESI) |  |
| --- | --- |
| calc. for C_23_H_32_N_5_O_3_S [M+H]^+^ | found |
| 458.2 | 458.9 |

**Supplementary Table 10:** Longitudinal analyses of factorial datasets for vehicle and JB253

| **Experiment** | **Statistic** | **df** | **P value** |
| --- | --- | --- | --- |
| Veh Dark | 19.80335 | 2.874937 | 2.149983e-12 |
| Veh Light | 23.33559 | 2.751172 | 4.797819e-14 |
| JB253 Dark | 5.892535 | 2.339218 | 1.554526e-03 |
| JB253 Light | 14.530117 | 2.346086 | 7.054928e-08 |

**Supplementary Figure 1:** Blood glucose levels cannot be reversibly controlled over the 20 min experimental window used in the present study (*n* = 7 animals). Data represent the mean ± S.E.M.

**Supplementary Figure 2:** Light does not significantly influence blood glucose levels following oral gavage with DMSO (*n* = 3 animals). NS, non-significant, repeated measures two-way ANOVA (Bonferroni’s posthoc). Data represent the mean ± S.E.M.


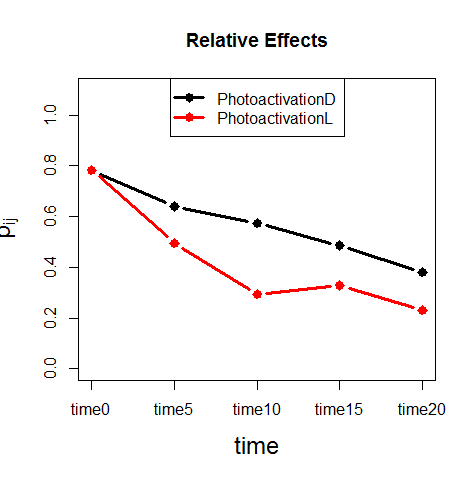


**Supplementary Figure 3:** Relative effects of dark (PhotoactivationD) and light (PhotoactivationL) on glycemia in JB253-treated animals.


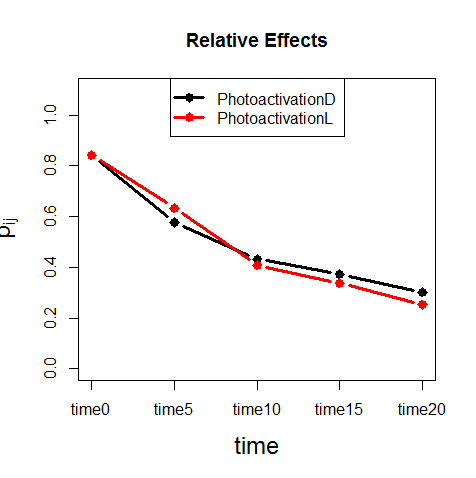


**Supplementary Figure 4:** Relative effects of dark (PhotoactivationD) and light (PhotoactivationL) on glycemia in vehicle-treated animals.

**
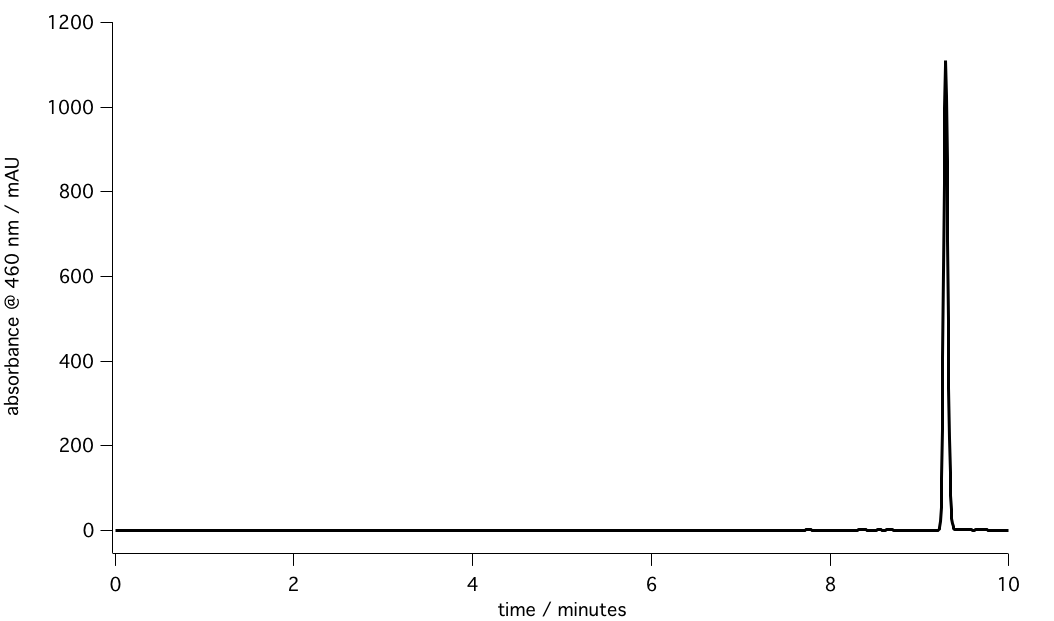
**

**Supplementary Figure 5:** LCMS-trace of JB253. *t_R_* = 9.280 min.
